# Associations of mucinous differentiation and mucin expression with immune cell infiltration and prognosis in colorectal adenocarcinoma

**DOI:** 10.1038/s41416-025-02960-3

**Published:** 2025-02-18

**Authors:** Hanna Elomaa, Vilma Tarkiainen, Ville K. Äijälä, Päivi Sirniö, Maarit Ahtiainen, Onni Sirkiä, Henna Karjalainen, Meeri Kastinen, Vilja V. Tapiainen, Jukka Rintala, Sanna Meriläinen, Juha Saarnio, Tero Rautio, Anne Tuomisto, Olli Helminen, Erkki-Ville Wirta, Toni T. Seppälä, Jan Böhm, Markus J. Mäkinen, Jukka-Pekka Mecklin, Juha P. Väyrynen

**Affiliations:** 1 Department of Education and Research, Hospital Nova of Central Finland, Well Being Services County of Central Finland, Jyväskylä, Finland; 2 Department of Biological and Environmental Science, University of Jyväskylä, Jyväskylä, Finland; 3 Translational Medicine Research Unit, University of Oulu, Medical Research Center Oulu, and Oulu University Hospital, Oulu, Finland; 4 Central Finland Biobank, Hospital Nova of Central Finland, Well Being Services County of Central Finland, Jyväskylä, Finland; 5 Department of Pathology, Hospital Nova of Central Finland, Well Being Services County of Central Finland, Jyväskylä, Finland; 6 Department of Environmental and Biological Sciences, University of Eastern Finland, Kuopio, Finland; 7 Department of Gastroenterology and Alimentary Tract Surgery, Tampere University Hospital, Tampere, Finland; 8 Faculty of Medicine and Health Technology, Tampere University and Tays Cancer Center, Tampere University Hospital, Tampere, Finland; 9 Department of Gastrointestinal Surgery, Helsinki University Hospital, Helsinki, Finland; 10 Applied Tumor Genomics Research Program, University of Helsinki, Helsinki, Finland; 11 Faculty of Sport and Health Sciences, University of Jyväskylä, Jyväskylä, Finland

**Keywords:** Colorectal cancer, Cancer microenvironment

## Abstract

**Background:**

The production of extracellular mucus and expression of mucins are commonly aberrant in colorectal cancer, yet their roles in tumour progression remain unclear.

**Methods:**

To investigate the potential influence of mucus on immune response and prognosis, we analysed mucinous differentiation (non-mucinous, 0%; mucinous component, 1–50%; mucinous, >50%) and its associations with immune cell densities (determined with three multiplex immunohistochemistry assays or conventional immunohistochemistry) and survival in 1049 colorectal cancer patients and a validation cohort of 771 patients. We also assessed expression patterns of transmembrane (MUC1, MUC4) and secreted (MUC2, MUC5AC and MUC6) mucins using immunohistochemistry.

**Results:**

Mucinous differentiation was associated with higher densities of CD14^+^HLADR^–^ immature monocytic cells and M2-like macrophages in mismatch repair (MMR) proficient tumours, and lower T-cell densities in MMR-deficient tumours. Mucinous differentiation was not associated with cancer-specific survival in multivariable Cox regression models. Higher cytoplasmic MUC1 expression independently predicted worse cancer-specific survival (multivariable HR for high vs. negative to low expression, 2.14; 95% CI: 1.26–3.64). It was also associated with increased myeloid cell infiltration in MMR-proficient tumours.

**Conclusions:**

Although mucinous differentiation did not independently predict survival, extracellular mucus and MUC1 expression could promote tumour progression through immunosuppression.

## Introduction

Colorectal cancer poses a significant global health burden, ranking as the third most common cancer worldwide [1]. It is a heterogeneous disease and its development is influenced by numerous genetic, molecular, and environmental alterations [2]. The epithelial surface of the large intestine is covered by a double mucus layer, playing crucial role in intestinal homoeostasis. Mucus serves to lubricate and protect the intestinal wall, acts as a selective barrier for small molecules, and shields against invading pathogens [3].

Mucins are O-glycosylated proteins with repetitive proline, threonine, and serine regions constituting the major protein component of mucus. They are categorised into transmembrane and secreted gel-forming mucins [4]. Transmembrane mucins like MUC1 and MUC4 anchor the mucus layer to the epithelium, forming a protective physical barrier and regulating cellular signal transduction [4, 5]. Secreted mucins such as MUC2, MUC5AC and MUC6 confer viscoelastic and chemical properties to mucus and create a highly hydrated gel to lubricate epithelial surfaces [4, 6]. However, chronic inflammation or colorectal carcinogenesis may lead to alterations in the mucus layer and mucin expression. Furthermore, aberrant expression of mucins may affect cancer development and growth, for instance, by altering signal transduction [7, 8]. Approximately 5–20% of all colorectal adenocarcinomas are categorised as mucinous adenocarcinomas, defined as carcinomas with extracellular mucus comprising over 50% of the lesion [9]. Compared to conventional adenocarcinomas, mucinous adenocarcinomas are more frequently located in the proximal colon, diagnosed at later stages, and associated with mismatch repair (MMR) deficiency [7]. Meta-analyses indicate that mucinous differentiation is associated with slightly worse prognosis in colorectal cancer [10, 11]. However, this association may be influenced by tumour molecular and histological features that were not evaluated in several studies included in the meta-analyses, such as MMR status.

Immune cells play a critical role in detecting and eliminating tumour cells, and higher immune cell infiltration is generally associated with favourable prognosis in colorectal cancer [12]. Immune cells have been proposed to interact with mucus by regulating its secretion or altering its composition [13]. Furthermore, a few studies have reported mucinous differentiation to be associated with immune cell infiltration or activation in colorectal cancer [14, 15], ovarian cancer [16] and gastric cancer [17], but comprehensive analyses of the associations of mucinous differentiation or mucin expression with the colorectal cancer immune microenvironment have not been conducted.

In this study, we identified mucinous differentiation and analysed immune cell infiltrates by three multiplex immunohistochemistry assays in a population-based colorectal cancer cohort of 1049 patients. Our primary aims were to (i) evaluate the associations of mucinous differentiation and immune cell infiltration in the tumour microenvironment and (ii) assess the prognostic value of mucinous differentiation. These aims were also studied in a validation cohort of 771 colorectal cancer patients. As secondary aims, we (iii) characterised the expression patterns of transmembrane mucins (MUC1 and MUC4) and secreted mucins (MUC2, MUC5AC, and MUC6) along with their prognostic value and associations with immune cell infiltration.

## Methods

### Study population

**Fig. 1:**
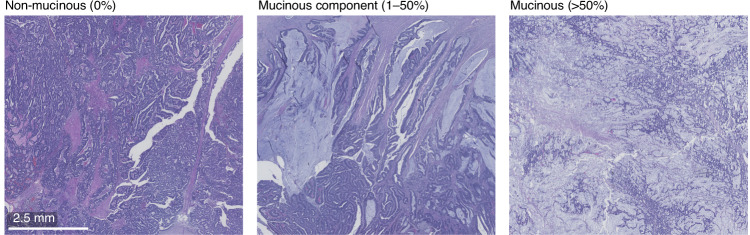
Mucinous differentiation in colorectal cancer. Examples of a non-mucinous tumour, a tumour with a mucinous component, and a mucinous tumour.

The study utilised a previously described cohort [18, 19] comprising 1343 patients who underwent primary colorectal cancer surgery between 2000 and 2015 at Central Finland Central Hospital. The clinical data were retrospectively collected from the hospital’s pathology registry. In addition, all tumours were previously re-evaluated for disease stage, tumour grade (WHO 2019 criteria), MMR status, *BRAF* V600E mutation status, and the presence of lymphovascular invasion [19]. The median follow-up time for censored cases was 10.3 years (IQR 6.9–13.3). Mucinous differentiation was evaluated using hematoxylin and eosin-stained whole slide sections by categorising tumours into non-mucinous tumours (0%), tumours with a mucinous component (>0–50%), and mucinous tumours (>50%) based on the fraction of extracellular mucus (Fig. 1). We used a three-tiered classification for mucinous differentiation to capture prognostic differences that may be present in tumours with a mucinous component below the standard >50% threshold for mucinous adenocarcinoma. To analyse mucin expression and immune cell infiltration with immunohistochemistry, four 1-mm diameter cores (two from both the tumour centre and the invasive margin) were inserted into 25 tissue microarray blocks [19]. Patients who received preoperative treatment including radiotherapy, chemotherapy, or chemoradiotherapy (*N* = 243) were excluded due to the potential impact on tumour characteristics [20]. In addition, patients who died within 30 days after surgery were excluded due to the possibility of postoperative complications (*N* = 30). Lastly, tumours with insufficient samples or unsuccessful immunohistochemistry for at least one type of mucin were also excluded (*N* = 21). The final cohort included 1049 patients (Table 1).

**Table 1:** Clinicopathologic characteristics of colorectal cancer patients stratified by mucinous differentiation.

		Mucinous differentiation			
Characteristic	Total *N*	0%	1–50%	>50%	*P*
All cases	1049 (100%)	668 (64%)	296 (28%)	84 (8%)	
Sex					0.032
Female	518 (49%)	310 (46%)	164 (55%)	44 (52%)	
Male	531 (51%)	358 (54%)	132 (45%)	41 (48%)	
Age (years)					0.055
<65	284 (27%)	198 (30%)	61 (21%)	25 (29%)	
65–75	369 (35%)	230 (34%)	109 (37%)	30 (35%)	
>75	396 (38%)	240 (36%)	126 (43%)	30 (35%)	
Year of operation					0.497
2000–2005	325 (31%)	215 (32%)	83 (28%)	27 (32%)	
2006–2010	334 (32%)	213 (32%)	99 (33%)	22 (26%)	
2011–2015	390 (37%)	240 (36%)	114 (39%)	36 (42%)	
Tumour location					<0.0001
Proximal colon	508 (48%)	265 (40%)	188 (64%)	55 (65%)	
Distal colon	386 (37%)	292 (44%)	72 (24%)	22 (26%)	
Rectum	155 (15%)	111 (17%)	36 (12%)	8 (9%)	
AJCC stage					0.327
I	177 (17%)	123 (18%)	44 (15%)	10 (12%)	
II	393 (37%)	249 (37%)	108 (37%)	36 (42%)	
III	343 (33%)	219 (33%)	99 (33%)	25 (29%)	
IV	136 (13%)	77 (12%)	45 (15%)	14 (17%)	
Tumour grade					0.001
Low-grade	868 (83%)	573 (86%)	233 (79%)	62 (73%)	
High -grade	181 (17%)	95 (14%)	63 (21%)	23 (27%)	
Lymphovascular invasion					0.080
No	828 (79%)	519 (78%)	234 (79%)	75 (88%)	
Yes	221 (21%)	149 (22%)	62 (21%)	10 (12%)	
MMR status					<0.0001
MMR-proficient	889 (85%)	619 (93%)	211 (71%)	59 (69%)	
MMR-deficient	160 (15%)	49 (7%)	85 (29%)	26 (31%)	
*BRAF* status^a^					<0.0001
Wild-type	880 (84%)	617 (93%)	206 (70%)	57 (67%)	
Mutant	168 (16%)	50 (8%)	90 (30%)	28 (33%)	

*AJCC* American Joint Committee on Cancer, *MMR* mismatch repair.

^a^Data missing for one case.

### Immunohistochemistry and image analysis for mucins

Immunohistochemistry for MUC1, MUC4, MUC2, MUC5AC, and MUC6 was performed on 3.5-µm thick sections using the Leica Bond III automated IHC stainer (Leica Biosystems, Buffalo Grove, IL, USA) and Bond Refine Detection kit (DS9800, Leica Biosystems). Chromogenic detection was done using 3’3-Diaminobenzidine (DAB) with hematoxylin (0.1%) for counterstaining. The antibodies and staining protocols for mucins are listed in Table S1. All antibodies and staining conditions were first validated using a test tissue microarray specimen consisting of 1.5-mm diameter cores with tonsil tissue, non-neoplastic colon, and a histologically diverse set of colorectal cancers. Immunohistochemistry-stained slides were coverslipped using Tissue-Tek Glas Automated Glass Coverslipper (Sakura Finetek, CA, USA) and digitised with a NanoZoomer-XR slide scanner (Hamamatsu Photonics, Hamamatsu City, Japan) with a ×20 objective. The staining patterns of each mucin in a representative case are presented in Fig. S1.

The expression patterns of mucins were visually analysed and did not show significant variation based on proximity to extracellular mucus. The expression of MUC1 and MUC4 membrane-bound mucins was assessed in the cytoplasm and apical cell membrane of tumour cells, while the expression of MUC2, MUC5AC, and MUC6 secreted mucins was evaluated in the cytoplasm. Mucin expression was assessed by evaluating the percentage of positive tumour cells with 5% intervals (0–100%) and the staining intensities were categorised as negative, low, moderate, or high. Examples of different cytoplasmic and membranous mucin expression levels and staining intensities are illustrated in Fig. S2. Subsequently, mucin histoscore was calculated as follows: histoscore = [(1× percentage of weakly stained cells) + (2× percentage of moderately stained cells) + (3× percentage of strongly stained cells)]. Each tumour core was individually analysed and in cases of multiple tissue microarray cores, the mean histoscore value was calculated across all tumour cores. All image analyses were conducted by two scientists blinded to associated data. For analyses, histoscore values were categorised into negative (0), low (> 0–90), intermediate (> 90–180), and high (> 180), as well as negative (0) and positive (> 0).

### Immune cell analyses

**Fig. 2:**
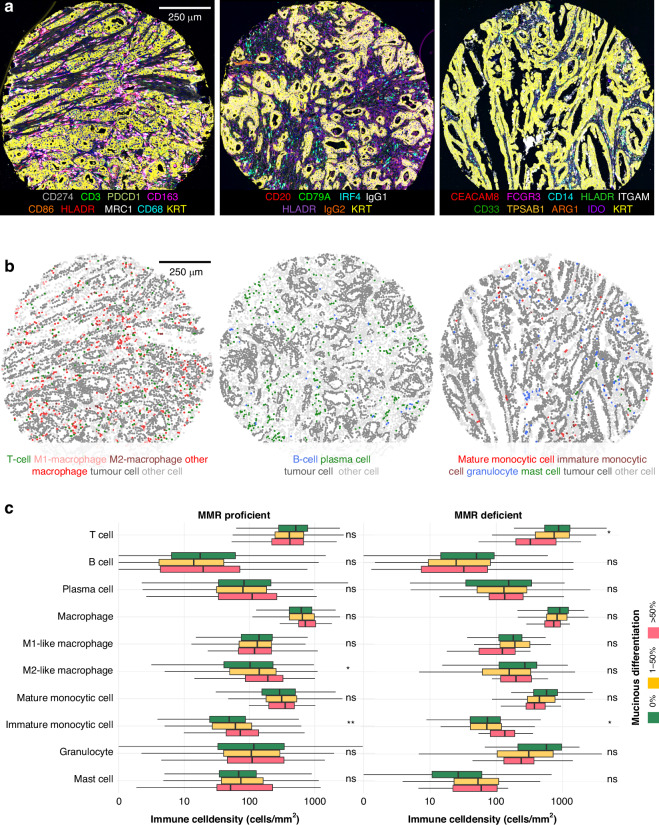
Immune cell phenotyping and associations between mucinous differentiation and immune cell densities. **a** pseudo-immunofluorescence images for visualising three multiplex immunohistochemistry staining panels. **b** Cell phenotyping maps for immune cell subtypes, tumour cells, and other cells. **c** Associations between mucinous differentiation and immune cell densities in MMR-proficient and deficient tumours. *P* values were determined using the Wilcoxon rank-sum test. Statistically significant correlations are shown with asterisks (***P < 0.0001; **P < 0.001; *P < 0.005). Immune cell density analyses for MMR-proficient and deficient tumours are based on 870 and 156 cases (T cells, macrophages, M1-like macrophages, M2-like macrophages), 873 and 159 cases (B cells, plasma cells), 856 and 152 cases (mature monocytic cells, immature monocytic cells, granulocytes, mast cells), respectively. MMR, mismatch repair.

The densities of multiple immune cell subtypes (cells/mm^2^) had been quantified in previous studies [21, 22] using cyclic multiplex immunohistochemistry assays. Cell densities were quantified in the tumour epithelial and stromal compartments. Mucinous areas were excluded because their minimal or absent immune cell content could impact the immune cell measurements. For this study, we included T cells (CD3^+^), macrophages (CD68^+^ and/or CD163^+^) [22], B cells (MS4A1^+^CD79A^+^), plasma cells (MS4A1^–^CD79A^+^), mature monocytic cells (CD14^+^HLADR^+^CEACAM8^–^TBSAB1^–^KRT^–^), immature monocytic cells (CD14^+^HLADR^+^CEACAM8^–^TBSAB1^–^KRT^–^), granulocytes (CEACAM8^+^CD14^–^TBSAB1^–^KRT^–^), and mast cells (TBSAB1^+^CD14^–^CEACAM8^–^KRT^–^) [21]. Macrophages were further phenotyped based on their polarisation state into M1-like and M2-like macrophages using a polarisation index that included marker intensities for M1-like macrophages (CD86 and HLADR) and M2-like macrophages (CD163 and MRC1), as previously described [22]. Examples of the multiplex immunohistochemistry staining panels and immune cell phenotyping are shown in Fig. 2a, b. The antibodies and staining protocols used for immune cell detection are listed in Table S2. Following the recommendations of the expert panel, we use the standardised nomenclature system for protein names [23].

### Statistical analyses

Statistical analyses were performed using RStudio (version 2023.06.2, RStudio Team) and R statistical programming (v.4.3.1, R Core Team) with the following packages: *corrplot* (0.92), *forestplot* (3.1.3), *ggplot2* (3.4.3), *ggpubr* (0.6.0), *gmodels* (2.18.1.1), *scales* (1.3.0), *survival* (3.5–5), *survminer* (0.4.9), and *tidyverse* (2.0.0). Cross-tabulation with Chi-square test was used to analyse the associations of categorical clinicopathological and histological data with mucin expression. Correlations between mucin expression and other variables were assessed using Spearman’s correlation coefficients. The associations between categorical data for mucinous differentiation/mucin expression and continuous data for immune cell densities were assessed using the Wilcoxon rank-sum test. For survival analyses, colorectal cancer-specific survival, defined as the period from surgery until cancer-related death, was the primary endpoint. Overall survival, defined as the time from surgery until death from any cause, was also analysed. The Kaplan–Meier method with a Log-rank test was used to visualise cancer-specific survival estimates. Univariable and multivariable Cox regression hazard models were conducted to analyse mortality hazard ratios (HRs) with 95% confidence intervals (CIs). Multivariable models included the following pre-determined covariates (with the reference category listed first): sex (male, female), age (< 65, 65–75, >75), year of operation (2000–2005, 2006–2010, 2011–2015), tumour location (proximal colon, distal colon, rectum), disease stage (I–II, III, IV), tumour grade (well/moderately differentiated, poorly differentiated), lymphovascular invasion (negative, positive), MMR status (proficient, deficient), *BRAF* V600E mutation status (wild-type, mutant), all known to influence the biology and survival in colorectal cancer. The survival analyses were further stratified by MMR status due to its associations with mucinous differentiation, as well as its role in influencing tumour behaviour and colorectal cancer patient survival. In Cox regression models of mucin expression, we combined mucin protein expression categories with fewer than 40 patients with the adjacent category to improve the stability of the multivariable models. Survival analyses were limited to 10 years, considering most deaths occurred within that time frame. All *P* values were two-sided and *P* value of <0.005 was considered statistically significant according to the recommendation of the expert panel [24].

### Validation cohort

Mucinous differentiation and its association with T-cell densities were analysed in an independent validation cohort of 1011 colorectal cancers to increase the reproducibility and generalisability of the findings [25]. The validation cohort was prospectively collected between years 2006 and 2020 in Oulu University Hospital. After excluding patients who had received any preoperative treatment (*N* = 235) or had died within 30 days after surgery (*N* = 5), the final cohort included 771 patients (Table S3). Data on T-cell densities were analysed using QuPath and were available for 754 patients [19]. The median follow-up time for censored cases was 6.1 years (IQR 3.9–9.9).

## Results

### Mucinous differentiation in colorectal cancer

Mucinous differentiation and the expression of mucins were analysed in 1049 colorectal cancers. Tumours were divided based on mucinous differentiation into non-mucinous tumours (0%), tumours with a mucinous component (1–50%), and mucinous tumours (> 50%). Of all tumours, 668 (64%) were non-mucinous, 296 (28%) contained a mucinous component of 1–50%, and 85 (8%) were mucinous in the main cohort (Table 1). In the validation cohort (*N* = 771), 67% were non-mucinous, 23% contained a mucinous component, and 10% were mucinous adenocarcinomas (Table S3). In both cohorts, mucinous differentiation was associated with proximal tumour location, high tumour grade, MMR deficiency and *BRAF* mutation (Table 1 and Table S3).

### Immune cell associations for mucinous differentiation

We examined the relationship between mucinous differentiation and immune cell densities. These analyses were conducted separately for MMR-deficient and MMR-proficient tumours, considering that MMR status is strongly associated with both mucinous differentiation and immune cell densities (Fig. 2c). Of MMR-proficient tumours, those with mucinous differentiation exhibited a higher density of M2-like macrophages compared to non-mucinous adenocarcinomas. In contrast, mucinous tumours with MMR deficiency showed lower T-cell infiltration compared to tumours with a mucinous component or non-mucinous tumours. In addition, regardless of MMR status, mucinous tumours displayed higher densities of CD14^+^HLADR^–^ immature monocytic cells. We further categorised images into tumour epithelial and stromal regions. In MMR-proficient tumours, the findings of higher densities of immature monocytic cells and M2-like macrophages remained in tumour epithelial compartment. In addition, mucinous differentiation was associated with higher density of intraepithelial mast cells and lower density of stromal T cells. In MMR-deficient tumours, the association with lower T-cell density remained in tumour epithelial compartment, and that of immature monocytic cells remained in both compartments. In addition, mucinous differentiation was associated with lower densities of macrophages and granulocytes (Fig. S3). The densities of B cells, plasma cells, M1-like macrophages, mature monocytic cells, granulocytes, or mast cells did not associate with mucinous differentiation. The finding of lower T-cell densities in MMR-deficient tumours that showed mucinous differentiation was confirmed in the validation cohort, and significant associations were found for both CD3^+^ T cells and CD8^+^ cytotoxic T cells (Fig. S4).

### Survival analyses for mucinous differentiation

**Fig. 3:**
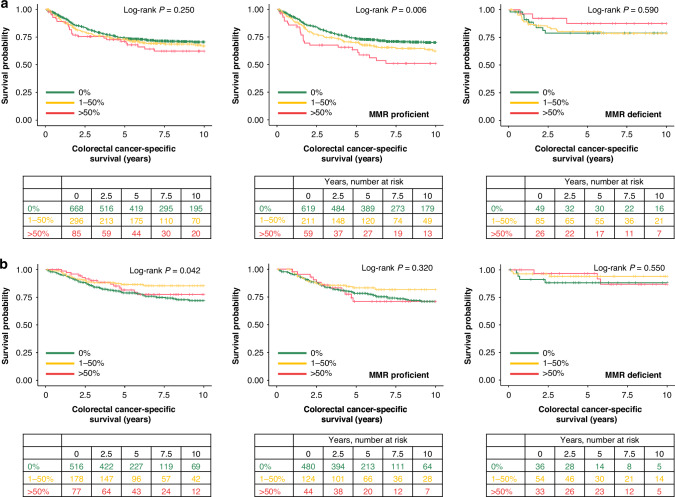
Kaplan–Meier estimates for cancer-specific survival. Kaplan–Meier survival curves for mucinous differentiation for all patients, patients with MMR-proficient tumours, and patients with MMR-deficient tumours in **a** the main cohort and **b** the validation cohort. MMR mismatch repair.

We assessed the prognostic value of mucinous differentiation. In the main cohort, there were a total of 611 (58%) deaths of which 301 (29%) were cancer-associated deaths. In the validation cohort, the total number of deaths was 264 (34%), including 146 (19%) cancer-associated deaths. Mucinous differentiation did not associate with cancer-specific or overall survival in either univariable (Fig. 3 and Table 2) or multivariable (Table 2) analyses in the main cohort. Complete multivariable survival models are presented in Table S4. We further examined the survival association of mucinous differentiation in strata of MMR status. In MMR-proficient tumours, mucinous differentiation was associated with poor colorectal cancer-specific survival in univariable analysis (*P*_trend_ = 0.002, HR for mucinous vs. non-mucinous 1.87, 95% CI 1.23–2.84 for cancer-specific survival) (Fig. 3 and Table 2), but the significance did not remain in multivariable analysis (Table 2). Similarly, in the validation cohort, mucinous differentiation did not demonstrate a prognostic role among all patients or patients with MMR-proficient or deficient tumours (Fig. 3 and Table S5). In addition, survival analyses using dichotomised groups of mucinous differentiation (≤ 50% mucinous component vs. >50% mucinous component) were performed for all tumours and stratified by MMR status, but no significant prognostic associations were observed in multivariable survival models (Table S6).

**Table 2:** Mucinous differentiation in relation to patient survival.

		Colorectal cancer-specific survival			Overall survival		
	No. of cases	No. of events	Univariable HR (95% CI)	Multivariable HR (95% CI)	No. of events	Univariable HR (95% CI)	Multivariable HR (95% CI)
All patients							
Mucinous differentiation	1049	292			525		
0%	668	178	1 (referent)	1 (referent)	317	1 (referent)	1 (referent)
1–50%	296	86	1.14 (0.88–1.48)	0.99 (0.76–1.30)	158	1.20 (0.99–1.45)	1.00 (0.82–1.22)
>50%	85	28	1.36 (0.91–2.02)	1.35 (0.89–2.04)	50	1.39 (1.03–1.87)	1.34 (0.98–1.82)
*P*_trend_			0.099	0.317		0.010	0.178
MMR-proficient							
Mucinous differentiation	889	263			444		
0%	619	169	1 (referent)	1 (referent)	292	1 (referent)	1 (referent)
1–50%	211	69	1.29 (0.97–1.70)	1.00 (0.75–1.33)	111	1.22 (0.98–1.51)	0.97 (0.78–1.22)
>50%	59	25	1.87 (1.23–2.84)	1.36 (0.88–2.11)	41	1.80 (1.30–2.50)	1.32 (0.94–1.85)
*P*_trend_			0.002	0.332		0.0004	0.311
MMR-deficient							
Mucinous differentiation	160	29			81		
0%	49	9	1 (referent)	1 (referent)	25	1 (referent)	1 (referent)
1–50%	85	17	1.01 (0.45–2.27)	1.34 (0.50–3.57)	47	1.04 (0.64–1.68)	1.00 (0.56–1.77)
>50%	26	3	0.54 (0.15–2.00)	1.55 (0.35–6.75)	9	0.61 (0.29–1.31)	0.93 (0.40–2.17)
*P*_trend_			0.442	0.506		0.304	0.887

*CI* confidence interval, *HR* hazard ratio, *MMR* mismatch repair.

Multivariable Cox proportional hazards regression models were adjusted for sex (male, female), age (< 65, 65–75, and >75 years), year of operation (2000–2005, 2006–2010, and 2011–2015), tumour location (proximal colon, distal colon, and rectum), stages (I–II, III and IV), tumour grade (low-grade or high-grade), lymphovascular invasion (negative or positive), MMR status (proficient or deficient), and *BRAF* status (wild-type or mutant). *P*_trend_ values were calculated by using the categories of mucinous differentiation as continuous variables in Cox regression models.

### Expression patterns of mucins

The expression patterns of five mucins were characterised by calculating histoscores based on staining intensity and the percentage of positive tumour cells. A total of 3582 tumour cores were successfully analysed (mean 3.4 per patient, SD 0.9). The expression of MUC1 and MUC4 membrane-bound mucins was detected in the cytoplasm (97% and 54% of all tumours, respectively) and in the apical membrane (91% and 60% of all tumours, respectively). Apical membranes were not visible in 7% of tumours analysed for MUC1 and in 6% of tumours analysed for MUC4. Expression of secreted mucins MUC2, MUC5AC, and MUC6 was detected in the cytoplasm (94%, 59% and 12% of all tumours, respectively). Core-to-core correlations for the expression of mucins in both the tumour centre and the invasive margin were moderate to high, indicating concordant expression of mucins across the whole tumour (Table S7). Mucin expression histoscores were grouped into four categories (from negative to high).

We analysed clinicopathological characteristics in relation to cytoplasmic mucin expression for both membrane-bound (Fig. S5) and secreted (Fig. S6) mucins. Consistent with the findings for mucinous differentiation, the expression of all mucins was increased in tumours with proximal colon location, high tumour grade, MMR deficiency, and *BRAF* mutation. In addition, higher expression of MUC1 and MUC2 was linked with later years of operation, and MUC2 expression was also associated with the absence of lymphovascular invasion. MUC5AC expression showed an increase in females and MUC6 in males.

We also assessed the relationship between mucinous differentiation (evaluated from hematoxylin and eosin-stained whole slide sections) and mucin expression (Fig. S7). Mucinous differentiation was positively correlated with the expression of all mucins, except for the membranous expression of MUC1. The strongest correlation was observed with cytoplasmic MUC2 expression (*R* = 0.63). Overall, the expression of various mucins was positively correlated, although cytoplasmic expression of MUC2 did not correlate with either cytoplasmic or membranous expression of MUC1.

### Survival analyses for mucins

We investigated the prognostic significance of mucins. For membrane-bound mucins MUC1 and MUC4, survival analyses were conducted for both cytoplasmic and membranous expression. Higher cytoplasmic expression of MUC1 was significantly associated with poor cancer-specific and overall survival in both univariable (Fig. S8 and Table S8) and multivariable (Table S8) analyses, independent of confounding factors such as stage, grade, and MMR status (*P*_trend_ = 0.0007, HR for high vs. negative to low 2.14, 95% CI 1.26–3.64). Further survival analyses for cytoplasmic MUC1 expression indicated that the prognostic significance did not significantly differ by MMR status (*P*_interaction_ ≥ 0.14 in univariable and *P*_interaction_ ≥ 0.46 in multivariable analyses) (Table S9). Contrary to cytoplasmic MUC1 expression, membranous MUC1 expression did not show a significant impact on survival (Fig. S9). Cytoplasmic MUC4 expression showed a tendency towards an association with favourable survival in univariable analysis (Fig. S8 and Table S8), but showed no significance in multivariable models (Table S8). Similar to MUC1, membranous MUC4 expression had no impact on survival (Fig. S9). The expression of secreted mucins MUC2, MUC5AC, and MUC6 demonstrated no significant prognostic associations in either univariable (Fig. S8 and Table S8) or multivariable analyses (Table S8).

### Immune cell associations for mucins

We examined the associations between cytoplasmic expression of mucins and immune cell densities separately in MMR-proficient and deficient tumours. In MMR-proficient tumours, higher expression of MUC1 was associated with increased densities of macrophages, mature and immature monocytic cells, and granulocytes. However, no such associations were found in MMR-deficient tumours (Fig. S10). The expression of other mucins, including MUC4 (Fig. S10), MUC2, MUC5AC, or MUC6 (Fig. S11), did not show statistically significant association with immune cell densities in either MMR-proficient or deficient tumours.

## Discussion

In this study, we characterised the impact of mucinous differentiation on immune cell infiltration in the colorectal cancer microenvironment, as well as its prognostic significance, utilising a main cohort of 1049 colorectal cancer cases and an independent validation cohort of 771 patients. In addition, we assessed the expression patterns and the roles of transmembrane mucins MUC1 and MUC4, and secreted mucins MUC2, MUC5AC, and MUC6. We found that mucinous differentiation did not independently predict cancer-specific or overall survival. It was associated with increased infiltration of immature monocytic cells, increased M2-like macrophage infiltration in MMR-proficient tumours, and decreased T-cell densities in MMR-deficient tumours. Among mucins, increased cytoplasmic expression of MUC1 served as an independent factor of unfavourable survival and was linked with increased myeloid cell infiltrates in MMR-proficient tumours. Taken together, these results, based on multiplex immunohistochemistry combined with digital image analysis to enable detailed immune cell phenotyping, suggest that mucinous differentiation is associated with an immunosuppressive microenvironment in colorectal cancer.

In colorectal cancer, the amount and composition of mucus is typically altered and may affect tumour progression [3, 6]. In our cohort, 36% of the tumours exhibited mucinous differentiation (> 0%). We found mucinous differentiation to be associated with proximal colon location, high tumour grade, and *BRAF* mutation, which are indications of a more aggressive tumour phenotype. Previous studies have also indicated that mucinous tumours have a distinct molecular origin, and they exhibit different biological and molecular features compared to non-mucinous tumours [7, 26, 27]. Some studies have reported mucinous differentiation or mucinous adenocarcinoma to be associated with unfavourable survival [28–30], whereas in some studies the association was dependent on the tumour location [31, 32] or stage [33]. However, several studies have not found a significant relationship between mucinous differentiation and survival in colorectal cancer [14, 34, 35]. In subgroup analyses, we analysed mucinous differentiation by MMR status, noting its higher prevalence in MMR-deficient tumours. While mucinous differentiation was linked to poor survival in MMR-proficient tumours in univariable analyses, the association did not persist in multivariable analyses. Overall, our findings and previous studies suggest limited prognostic value of mucinous differentiation in colorectal cancer.

High infiltration of immune cells within the colorectal cancer microenvironment typically indicates an immunologically active tumour and predicts favourable survival and better response to immunotherapy. However, certain immune cells may possess anti-inflammatory and tumour growth-promoting effects [12]. We found that both MMR-proficient and deficient tumours with mucinous differentiation had increased densities of immature monocytic cells. In addition, mucinous differentiation was associated with a higher density of M2-like macrophages in MMR-proficient tumours and with a lower density of T cells in MMR-deficient tumours. High infiltration of T cells, particularly cytotoxic T cells is acknowledged as a strong favourable prognostic factor in colorectal cancer [36], while both immature monocytic cells and M2-like macrophages have been associated with disease progression [37, 38]. M2-like macrophages are mature monocytic cells known for their tendency to support tumour growth by dampening anti-tumour immune responses [39]. However, cancer can also trigger aberrant immature myeloid cell generation in the bone marrow and release into circulation and peripheral tissues. These cells are often referred to as myeloid-derived suppressor cells, possessing potent tumour growth-promoting and immunosuppressive capabilities [40]. Therefore, our results suggest that mucinous differentiation could promote tumour growth by upregulating immunosuppressive immune cells and by depleting the infiltration of T cells.

The interaction between mucus and the immune microenvironment has been incompletely understood, and previous studies have been limited by the lack of stratification by MMR status, as well as the qualitative or single-marker methods used for cell type identification. Notably, accurate identification of many myeloid cells requires multimarker analysis, which is not possible using conventional immunohistochemistry with a single chromogen. Immune cell infiltration patterns differ significantly between mucinous and non-mucinous tumours across various cancer types, reflecting distinct tumour microenvironment characteristics. In colorectal cancer, lower densities of tumour-infiltrating lymphocytes have been observed in mucinous compared to non-mucinous subtypes [15]. Similarly, in gastric cancer, mucinous tumours exhibit reduced T-cell infiltration relative to intestinal-type tumours, with T cells predominantly localised at the periphery rather than within tumour nests, suggesting an immune-excluded phenotype. Macrophage infiltration in tumour nests is also lower in mucinous gastric tumours than in intestinal or diffuse subtypes [17]. In mucinous ovarian cancer, the majority of tumours were described as immune cold, characterised by the absence of T cells and CD274^+^ cells [16]. However, contrasting findings have been reported as one study observed higher densities of tumour-infiltrating lymphocytes in mucinous colorectal cancers regardless of MMR status [14], while another found no significant differences in immune cell composition between mucinous and non-mucinous colorectal cancers [26].

Several factors may explain weaker immune infiltrates in mucinous tumours. Extracellular mucus may create a physical barrier or hinder immune cell infiltration [15]. It could also disrupt T-cell migration, tumour recognition and activation, or limit antitumor activity [17, 41]. Furthermore, the absence or reduced levels of T-cell-attracting chemokines in the mucinous tumour microenvironment could account for the decreased amount of T cells within tumour nests [42]. Our results add to these hypotheses by showing increased densities of immunosuppressive myeloid cells (M2-like macrophages and immature monocytic cells) in tumours with mucinous differentiation that could contribute to immune depletion.

Mucin proteins form the mucus layer of the large intestine, primarily composed of MUC2 [4, 5, 7], along with other mucins like MUC1 and MUC4. [6]. In contrast, gastric mucins MUC5AC and MUC6 are typically absent in the non-neoplastic large intestine [5]. In carcinogenesis, mucins may lose the tissue specificity and their expression levels are typically altered [7, 43]. We found positive correlations between mucinous differentiation and the expression of all five mucins, except for the membranous MUC1 expression, indicating higher mucin levels in mucinous versus non-mucinous tumours. Clinicopathological associations of mucins were mostly similar to those of mucinous differentiation, suggesting that increased mucin levels correlate with enhanced mucus production in colorectal cancer. Higher cytoplasmic MUC1 expression predicted unfavourable survival, independently of confounding factors, consistent with previous studies [44–48]. Among mucins, only MUC1 showed significant associations with immune cell infiltration. Higher MUC1 expression associated with increased infiltration of macrophages, mature and immature monocytic cells, and granulocytes in MMR-proficient tumours.

To our knowledge, this is the first study examining the relationships between mucinous differentiation, mucin expression, and the infiltration of detailed immune cell populations defined with multiplex immunohistochemistry. However, some limitations need to be considered in the interpretation of these results. First, the expression of mucins, as well as immune cell densities, were analysed from tissue microarrays, which may not fully represent the milieu of the whole tumour [49]. However, multiple tumour cores were analysed for each patient (mean 3.4 for mucin analyses), increasing the validity of the analysis. Second, some of the associations between mucinous differentiation/mucin expression and immune cell densities were relatively weak. However, the finding of lower T-cell densities in MMR-deficient tumours that displayed mucinous differentiation was validated in an independent cohort, strengthening the reliability of this result. Third, the expression of mucins was visually analysed, which might be less reproducible than automated analysis. However, to increase the accuracy, we analysed both the expression intensity and expression level using a histoscore method and used loose cut-off points to categorise mucin expression for the analyses. Furthermore, the expression of transmembrane mucins MUC1 and MUC4 was analysed separately in the cytoplasm and the apical membrane. Fourth, excluding all patients with preoperative treatment from analyses due to their potential effect on the immune microenvironment led to an underrepresentation of rectal cancers in the study cohorts and may have biased the cohort towards early-stage tumours, possibly diminishing the observed effect of mucinous differentiation on survival outcomes. Fifth, data on tumour deposits and extramural vascular invasion were not available. However, we adjusted multivariable survival models for several other important histopathological factors, including disease stage, tumour grade, and lymphovascular invasion. Sixth, this study included a high number of comparisons. However, to mitigate the risk of false positive findings, we used a strict *P* value threshold (0.005) as recommended by an expert panel [24]. However, this study includes important strengths. It was based on a large study cohort of 1049 colorectal cancers and an independent validation cohort of 771 tumours with comprehensive clinical and pathological data. Mucinous differentiation of the tumours was uniformly analysed from hematoxylin and eosin-stained whole slide specimens. Immune cell analyses were conducted using multiplex immunohistochemistry and machine learning-based image analysis, which enabled detailed immune cell phenotyping uniformly across all tumours.

In conclusion, mucinous differentiation did not predict survival in colorectal cancer, but its associations with immune cell infiltration suggest that increased levels of mucus could foster tumour progression by inducing an immunosuppressive microenvironment. Furthermore, MUC1 could contribute to the tumour growth-promoting role of mucus and serve as an independent indicator of poor survival in colorectal cancer.

## Supplementary information

Supplementary Online Material

The REMARK checklist

## Data Availability

The data generated and/or analysed during this study are not publicly available. The sharing of data will require approval from relevant ethics committees and/or biobanks. Further information including the procedures to obtain and access data of Finnish Biobanks are described at https://finbb.fi/en/fingenious-service.
