# Supplementary material for: Associations of mucinous differentiation and mucin expression with immune cell infiltration and prognosis in colorectal adenocarcinoma
Source: Br J Cancer. 2025 Feb 18;132(7):660–9. doi: 10.1038/s41416-025-02960-3 (PMC11961615; doi:10.1038/s41416-025-02960-3)
Supplement: Supplementary file 1 — Supplementary Online Material [file 41416_2025_2960_MOESM1_ESM.pdf]

Supplementary Table S1. Antibodies and staining protocols for mucins.

Antibody	Clone	Manufacturer	Catalogue number	Dilution	Secreted/ transmembrane	Host species
MUC1	GP1.4	Leica	PA0035	ready-to-use	transmembrane	mouse
MUC4	D4W9X	Cell Signaling	81692	1:250	transmembrane	rabbit
MUC2	CCP58	Leica	PA0155	ready-to-use	secreted	mouse
MUC5AC	CLH2	Leica	PA0052	ready-to-use	secreted	mouse
MUC6	CLH5	Leica	PA0053	ready-to-use	secreted	mouse

Heat-induced epitope retrieval was done using BOND epitope retrieval solution 2 (EDTA-based, pH 9.0, AR9640, Leica Biosystems) with 20 minutes heating time. The incubation time for primary antibodies was 30 minutes.

Supplementary Table S2. Antibodies and staining protocols included in multiplex immunohistochemistry staining panels 1–3 to phenotype immune cells and tumor cells.

Antibody	Clone	Manufacturer	Catalogue number	Epitope retrieval	Dilution	Host species
**Panel 1**						
CD274	E1L3N	Cell Signaling	13684S	ER2, 30 min	1:100	rabbit
CD3	LN10	Leica	PA055	ER1, 60 min	ready-to-use	mouse
PDCD1	SP269	Abcam	ab227681	ER1, 30 min	1:40	rabbit
CD163	10D6	Thermo Fisher Scientific	MS-1103	ER1, 60 min	1:100	mouse
CD86	E2G8P	Cell Signaling	91882S	ER1, 30 min	1:50	rabbit
HLADR	TAL 1B5	Santa Cruz	sc-53319	ER1, 60 min	1:4000	mouse
MRC1	E2L9N	Cell Signaling	91992S	ER1, 30 min	1:500	rabbit
CD68	KP1	Biolegend	916104	ER1, 30 min	1:40 000	mouse
KRT	BS5	BioSite Histo	BSH-7124-1	ER1, 60 min	1:400	mouse
**Panel 2**						
MS4A1	L26	Diagnostic Biosystems	Mob004	ER2, 20 min	1:100	mouse
CD79A	SP18	Thermo Fisher Scientific	RM-9118	ER1, 60 min	1:600	rabbit
IRF4	MUM1p	Dako	M7259	ER1, 60 min	1:200	mouse
IGHG1	RM117	RevMAb	31-1019-00	ER1, 60 min	1:4000	rabbit
HLADR	TAL 1B5	Santa Cruz	sc-53319	ER1, 60 min	1:4000	mouse
IGHG2	EPR4418	Abcam	ab134050	ER1, 60 min	1:40 000	rabbit
IGHG4	HP6025	Thermo Fisher Scientific	605-900	ER1, 60 min	1:500	mouse
KRT	BS5	BioSite Histo	BSH-7124-1	ER1, 60 min	1:400	mouse
**Panel 3**						
CEACAM8	G10F5	BioLegend	305102	ER2, 20 min	1:100	mouse
FCGR3	D1N9L	Cell Signaling	24326S	ER1, 30 min	1:70	rabbit
CD14	D7A2T	Cell Signaling	75181S	ER1, 60 min	1:70	rabbit
HLADR	TAL 1B5	Santa Cruz	sc-53319	ER1, 60 min	1:4000	mouse
ITGAM	D6X1N	Cell Signaling	49420S	ER1, 30 min	1:60	rabbit
CD33	SP266	Abcam	ab199432	ER1, 60 min	1:500	rabbit
TBSAB1	AA1	Santa Cruz	sc-59587	ER1, 60 min	1:8000	mouse
ARG1	EPR6672(B)	Abcam	ab133543	ER1, 30 min	1:500	rabbit
IDO	EPR20374	Abcam	ab211017	ER1, 60 min	1:500	rabbit
KRT	BS5	BioSite Histo	BSH-7124-1	ER1, 60 min	1:400	mouse

Primary antibodies used in the multiplex immunohistochemistry panels 1–3 were used to phenotype T cells, all macrophages, M1-like macrophages, and M2-like macrophages (Panel 1), B cells and plasma cells (Panel 2), and granulocytes, mature monocytic cells, immature monocytic cells, and granulocytes (Panel 3). The incubation time for primary antibodies was 30 minutes, except for 60 min for PDCD1. Heat-induced epitope retrieval was done using BOND epitope retrieval solution 1 (ER1, citrate-based, pH 6.0, AR9961, Leica Biosystems) or 2 (ER2, EDTA-based, pH 9.0, AR9640, Leica Biosystems) with 20–60 minutes heating time.

Supplementary Table S3. Clinicopathologic characteristics of colorectal cancer patients stratified by mucinous differentiation in the validation cohort.

		Mucinous differentiation			
Characteristic	Total N	0%	1–50%	>50%	*P*
All cases	771 (100%)	516 (67%)	178 (23%)	77 (10%)	
Sex					0.0083
Female	363 (47%)	223 (43%)	96 (54%)	44 (57%)	
Male	408 (53%)	293 (57%)	82 (46%)	33 (43%)	
Age (years)					0.42
<65	233 (30%)	150 (29%)	58 (33%)	25 (32%)	
65–75	284 (37%)	202 (39%)	59 (33%)	23 (30%)	
>75	254 (33%)	164 (32%)	61 (34%)	29 (38%)	
Year of operation					0.014
2006–2010	153 (20%)	99 (19%)	40 (22%)	14 (18%)	
2011–2015	216 (28%)	129 (25%)	55 (31%)	32 (42%)	
2016–2020	402 (52%)	288 (56%)	83 (47%)	31 (40%)	
Tumor location					<0.0001
Proximal colon	320 (42%)	160 (31%)	107 (60%)	53 (69%)	
Distal colon	204 (26%)	161 (31%)	29 (16%)	14 (18%)	
Rectum	247 (32%)	195 (38%)	42 (24%)	10 (13%)	
AJCC stage					0.0057
I	186 (24%)	115 (22%)	58 (33%)	13 (17%)	
II	253 (33%)	158 (31%)	63 (35%)	32 (42%)	
III	248 (32%)	180 (35%)	45 (25%)	23 (30%)	
IV	84 (11%)	63 (12%)	12 (6.7%)	9 (12%)	
Tumor grade					<0.0001
Low-grade	662 (86%)	452 (88%)	157 (88%)	53 (69%)	
High-grade	109 (14%)	64 (12%)	21 (12%)	24 (31%)	
Lymphovascular invasion					0.020
No	427 (55%)	271 (53%)	103 (58%)	53 (69%)	
Yes	344 (45%)	245 (47%)	75 (42%)	24 (31%)	
MMR status					<0.0001
MMR proficient	648 (84%)	480 (93%)	124 (70%)	44 (57%)	
MMR deficient	123 (16%)	36 (7.0%)	54 (30%)	33 (43%)	
BRAF status[1]					<0.0001
Wild-type	659 (86%)	473 (93%)	135 (76%)	51 (66%)	
Mutant	105 (14%)	37 (7%)	42 (24%)	26 (34%)	

Abbreviations: AJCC, American Joint Committee on Cancer; MMR, mismatch repair.
[1]*BRAF* data missing for 7 patients.

Supplementary Table S4. Multivariable Cox regression models for mucinous differentiation and cytoplasmic expression of MUC1 and patient survival.

	Mucinous differentiation		Cytoplasmic MUC1 expression	
	Cancer-specific survival	Overall survival	Cancer-specific survival	Overall survival
	Multivariable HR (95% CI)	Multivariable HR (95% CI)	Multivariable HR (95% CI)	Multivariable HR (95% CI)
Mucinous differentiation			Not included	Not included
Non-mucinous (0%)	1 (referent)	1 (referent)		
Mucinous component (1–50%)	0.99 (0.76-1.30)	1.00 (0.82-1.22)		
Mucinous (>50%)	1.35 (0.89-2.04)	1.34 (0.98-1.82)		
MUC1 expression				
Negative to low	Not included	Not included	1 (referent)	1 (referent)
Moderate			1.46 (1.09-1.96)	1.29 (1.03-1.62)
High			2.14 (1.26-3.64)	1.92 (1.27-2.89)
Age				
<65	1 (referent)	1 (referent)	1 (referent)	1 (referent)
65–75	1.16 (0.86-1.57)	1.35 (1.05-1.74)	1.13 (0.84-1.52)	1.32 (1.03-1.69)
>75	1.87 (1.39-2.52)	3.01 (2.39-3.80)	1.85 (1.38-2.47)	2.97 (2.36-3.75)
Sex				
Male	1 (referent)	1 (referent)	1 (referent)	1 (referent)
Female	0.83 (0.65-1.06)	0.74 (0.62-0.89)	0.84 (0.66-1.07)	0.74 (0.62-0.88)
Year of operation				
2000–2005	1 (referent)	1 (referent)	1 (referent)	1 (referent)
2006–2010	0.62 (0.47-0.83)	0.70 (0.56-0.86)	0.60 (0.45-0.80)	0.68 (0.55-0.84)
2011–2015	0.48 (0.36-0.65)	0.60 (0.48-0.74)	0.43 (0.31-0.58)	0.55 (0.43-0.69)
Tumor location				
Proximal colon	1 (referent)	1 (referent)	1 (referent)	1 (referent)
Distal colon	0.87 (0.67-1.14)	0.94 (0.77-1.16)	0.91 (0.70-1.19)	0.96 (0.79-1.18)
Rectum	0.80 (0.55-1.15)	0.88 (0.66-1.16)	0.83 (0.57-1.20)	0.89 (0.67-1.17)
AJCC stage				
I–II	1 (referent)	1 (referent)	1 (referent)	1 (referent)
III	3.03 (2.20-4.16)	1.48 (1.20-1.82)	3.04 (2.21-4.19)	1.49 (1.21-1.83)
IV	18.3 (13.0-25.7)	7.99 (6.23-10.2)	18.1 (12.9-25.4)	7.88 (6.16-10.1)
Tumor grade				
Low-grade	1 (referent)	1 (referent)	1 (referent)	1 (referent)
High-grade	1.77 (1.30-2.41)	1.84 (1.46-2.32)	1.61 (1.18-2.21)	1.68 (1.32-2.15)
Lymphovascular invasion				
No	1 (referent)	1 (referent)	1 (referent)	1 (referent)
Yes	2.00 (1.55-2.58)	1.66 (1.35-2.03)	1.94 (1.51-2.49)	1.61 (1.31-1.97)
MMR status				
MMR proficient	1 (referent)	1 (referent)	1 (referent)	1 (referent)
MMR deficient	0.52 (0.31-0.88)	0.65 (0.46-0.92)	0.49 (0.30-0.81)	0.62 (0.44-0.87)
BRAF mutation				
Wild-type	1 (referent)	1 (referent)	1 (referent)	1 (referent)
Mutant	1.30 (0.85-2.00)	1.43 (1.05-1.96)	1.27 (0.84-1.92)	1.45 (1.07-1.95)

Abbreviations: AJCC, American Joint Committee on Cancer; CI, confidence interval; HR, hazard ratio; MMR, mismatch repair.

Supplementary Table S5. Mucinous differentiation and patient survival for all patients and in strata of MMR status in the validation cohort.

		Colorectal cancer-specific survival			Overall survival		
	No. of cases	No. of events	Univariable HR (95% CI)	Multivariable HR (95% CI)	No. of events	Univariable HR (95% CI)	Multivariable HR (95% CI)
**All patients**							
Mucinous differentiation	771	146			264		
0%	516	109	1 (referent)	1 (referent)	179	1 (referent)	1 (referent)
1–50%	178	23	0.57 (0.36-0.90)	0.81 (0.50-1.31)	53	0.77 (0.56-1.04)	0.84 (0.60-1.18)
>50%	77	14	0.79 (0.45-1.38)	0.83 (0.46-1.49)	32	1.07 (0.74-1.57)	0.99 (0.65-1.50)
P_{trend}			0.066	0.37		0.64	0.67
**MMR proficient**							
Mucinous differentiation	648	136			222		
0%	480	105	1 (referent)	1 (referent)	167	1 (referent)	1 (referent)
1–50%	124	20	0.70 (0.44-1.13)	0.84 (0.51-1.38)	36	0.77 (0.54-1.10)	0.83 (0.57-1.21)
>50%	44	11	1.07 (0.58-1.99)	0.85 (0.45-1.61)	19	1.15 (0.72-1.86)	1.16 (0.71-1.89)
P_{trend}			0.54	0.45		0.75	0.97
**MMR deficient**							
Mucinous differentiation	123	10			42		
0%	36	4	1 (referent)	*	12	1 (referent)	1 (referent)
1–50%	54	3	0.44 (0.10-1.98)		17	0.74 (0.35-1.56)	0.86 (0.37-2.02)
>50%	33	3	0.71 (0.16-3.19)		13	0.93 (0.42-2.05)	0.76 (0.33-1.78)
P_{trend}			0.63			0.89	0.53

Multivariable Cox proportional hazards regression models were adjusted for sex (male, female), age (<65, 65–75, and >75 years), year of operation (2006–2010, 2011–2015, and 2016–2020), tumor location (proximal colon, distal colon, and rectum), stages (I–II, III, and IV), tumor grade (low-grade, high-grade), lymphovascular invasion (negative or positive), MMR status (proficient or deficient), and *BRAF* status (wild type or mutant). P_{trend} values were calculated by using the categories of mucinous differentiation as continuous variables. Abbreviations: CI, confidence interval; HR, hazard ratio; MMR, mismatch repair.

* No multivariable model presented because of the small number of events (N=10) leading to an unstable model.

Supplementary Table S6. Patient survival for non-mucinous (≤50% of extracellular mucus) and mucinous (>50% of extracellular mucus) tumor types for all patients and stratified by MMR status.

		Colorectal cancer-specific survival			Overall survival		
	No. of cases	No. of events	Univariable HR (95% CI)	Multivariable HR (95% CI)	No. of events	Univariable HR (95% CI)	Multivariable HR (95% CI)
**All patients**							
Tumor type	1049	292			525		
non-mucinous	964	264	1 (referent)	1 (referent)	475	1 (referent)	1 (referent)
mucinous	85	28	1.30 (0.88-1.92)	1.35 (0.91-2.02)	50	1.31 (0.98-1.76)	1.34 (0.99-1.80)
P			0.182	0.140		0.068	0.055
**MMR proficient**							
Tumor type	889	263			444		
non-mucinous	830	238	1 (referent)	1 (referent)	403	1 (referent)	1 (referent)
mucinous	59	25	1.75 (1.16-2.64)	1.36 (0.89-2.09)	41	1.72 (1.24-2.37)	1.33 (0.95-1.85)
P			0.008	0.159		0.001	0.094
**MMR deficient**							
Tumor type	160	29			160		
non-mucinous	134	26	1 (referent)	1 (referent)	134	1 (referent)	1 (referent)
mucinous	26	3	0.54 (0.16-1.78)	1.27 (0.35-4.69)	26	0.60 (0.30-1.19)	0.93 (0.44-1.99)
P			0.309	0.718		0.145	0.857

Multivariable Cox proportional hazards regression models were adjusted for sex (male, female), age (<65, 65–75, and >75 years), year of operation (2006–2010, 2011–2015, and 2016–2020), tumor location (proximal colon, distal colon, and rectum), stages (I–II, III, and IV), tumor grade (low-grade, high-grade), lymphovascular invasion (negative or positive), MMR status (proficient or deficient), and *BRAF* status (wild type or mutant). Abbreviations: CI, confidence interval; HR, hazard ratio; MMR, mismatch repair.

Supplementary Table S7. Core-to-core correlation of mucin expression in the tumor center and the invasive margin.

Mucin expression	*R* (tumor center)	N (tumor center)	*R* (invasive margin)	N (invasive margin)
MUC1 cytoplasm	0.61	1243	0.59	958
MUC1 membrane	0.59	1100	0.45	784
MUC4 cytoplasm	0.62	1220	0.65	907
MUC4 membrane	0.59	1067	0.57	756
MUC2 cytoplasm	0.67	1242	0.60	975
MUC5AC cytoplasm	0.58	1246	0.59	971
MUC6 cytoplasm	0.57	1218	0.62	929

Spearman's rank correlation coefficients (*R*) are used to calculate correlations in two randomly chosen cores of tumors with two or more cores (N).

Supplementary Table S8. Cytoplasmic expression of mucins in relation to patient survival.

	Colorectal cancer-specific survival				Overall survival		
	No. of cases	No. of events	Univariable HR (95% CI)	Multivariable HR (95% CI)	No. of events	Univariable HR (95% CI)	Multivariable HR (95% CI)
MUC1 expression	1049	292			525		
Negative to low	798	205	1 (referent)	1 (referent)	379	1 (referent)	1 (referent)
Moderate	209	69	1.51 (1.15-1.98)	1.46 (1.09-1.96)	116	1.41 (1.15-1.74)	1.29 (1.03-1.62)
High	42	18	2.30 (1.42-3.73)	2.14 (1.26-3.64)	30	2.33 (1.61-3.39)	1.92 (1.27-2.89)
P_{trend}			<0.0001	0.0007		<0.0001	0.0007
MUC2 expression	1049	292			525		
Negative	60	20	1 (referent)	1 (referent)	35	1 (referent)	1 (referent)
Low	735	203	0.72 (0.45-1.14)	0.79 (0.49-1.26)	355	0.74 (0.52-1.05)	0.87 (0.61-1.23)
Moderate	78	22	0.75 (0.41-1.37)	0.68 (0.37-1.27)	40	0.80 (0.51-1.26)	0.72 (0.45-1.15)
High	176	47	0.71 (0.42-1.20)	1.06 (0.62-1.82)	95	0.84 (0.57-1.24)	1.08 (0.72-1.60)
P_{trend}			0.583	0.378		0.663	0.328
MUC4 expression	1049	292			525		
Negative	484	116	1 (referent)	1 (referent)	224	1 (referent)	1 (referent)
Low	525	166	1.43 (1.13-1.81)	1.30 (1.01-1.66)	280	1.28 (1.08-1.53)	1.21 (1.01-1.46)
Moderate to high	40	10	1.14 (0.60-2.18)	0.99 (0.50-1.96)	21	1.24 (0.79-1.94)	0.88 (0.55-1.42)
P_{trend}			0.013	0.12		0.009	0.211
MUC5AC expression	1049	292			525		
Negative	433	126	1 (referent)	1 (referent)	216	1 (referent)	1 (referent)
Low	503	136	0.93 (0.73-1.19)	0.80 (0.62-1.03)	243	0.98 (0.82-1.18)	0.87 (0.72-1.06)
Moderate	52	16	1.17 (0.70-1.98)	1.16 (0.67-2.02)	30	1.27 (0.87-1.86)	1.13 (0.75-1.70)
High	61	14	0.86 (0.50-1.50)	0.98 (0.53-1.83)	36	1.32 (0.93-1.88)	1.13 (0.75-1.70)
P_{trend}			0.720	0.576		0.131	0.901
MUC6 expression	1049	292			525		
Negative	920	262	1 (referent)	1 (referent)	449	1 (referent)	1 (referent)
Low to high	129	30	0.85 (0.58-1.24)	1.00 (0.64-1.55)	76	1.28 (1.01-1.64)	1.35 (1.01-1.82)
P_{trend}			0.408	0.991		0.045	0.045

Multivariable Cox proportional hazards regression models were adjusted for sex (male, female), age (<65, 65–75, and >75 years), year of operation (2000–2005, 2006–2010, and 2011–2015), tumor location (proximal colon, distal colon, and rectum), stages (I–II, III, and IV), tumor grade (low-grade or high-grade), lymphovascular invasion (negative or positive), MMR status (proficient or deficient), and *BRAF* status (wild type or mutant). Mucin expression categories with fewer than 40 patients were combined with the adjacent ordinal category to enhance the stability of the multivariable regression models. P_{trend} values were calculated by using the categories of mucin expression as continuous variables in Cox regression models. Abbreviations: CI, confidence interval; HR, hazard ratio.

Supplementary Table S9. Cytoplasmic expression of MUC1 and patient survival in strata of MMR status.

		Colorectal cancer-specific survival			Overall survival		
	No. of cases	No. of events	Univariable HR (95% CI)	Multivariable HR (95% CI)	No. of events	Univariable HR (95% CI)	Multivariable HR (95% CI)
**MMR proficient**							
MUC1 expression	889	263			444		
Negative or low	709	189	1 (referent)	1 (referent)	335	1 (referent)	1 (referent)
Moderate or high	180	74	1.95 (1.49-2.55)	1.58 (1.17-2.12)	109	1.69 (1.36-2.10)	1.35 (1.06-1.72)
P			<0.0001	0.003		<0.0001	0.016
**MMR deficient**							
MUC1 expression	160	29			81		
Negative or low	89	16	1 (referent)	1 (referent)	44	1 (referent)	1 (referent)
Moderate or high	71	13	1.10 (0.53-2.28)	0.75 (0.30-1.88)	37	1.17 (0.75-1.81)	1.12 (0.67-1.86)
P			0.807	0.536		0.488	0.672
$P_{interaction}$			0.168	0.463		0.138	0.654

The expression of MUC1 was divided into negative or low and moderate or high. Multivariable Cox proportional hazards regression models were adjusted for sex (male, female), age (<65, 65–75, >75), year of operation (2000–2005, 2006–2010, 2011–2015), tumor location (proximal colon, distal colon, rectum), stage (I–II, III, IV), tumor grade (low-grade, high-grade), lymphovascular invasion (negative, positive), and *BRAF* status (wild-type, mutant). $P_{interaction}$ was calculated using the Wald test for the cross product of the MUC1 expression and MMR status in the Cox regression model. Abbreviations: CI, confidence interval; HR, hazard ratio; MMR, mismatch repair.

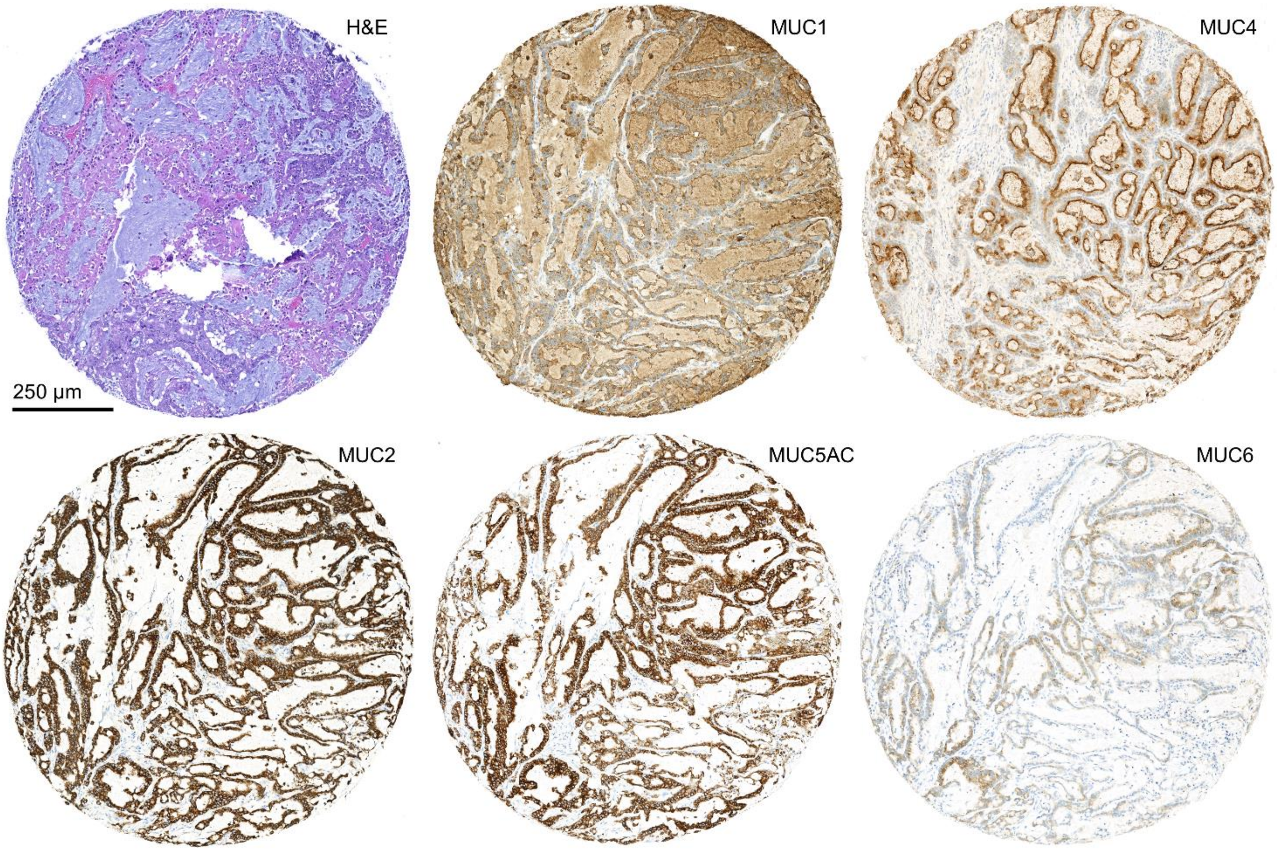

Figure S1. Expression patterns of mucin proteins. Example of a mucinous tumor core stained with Hematoxylin and Eosin (H&E), along with immunohistochemical staining for mucins.

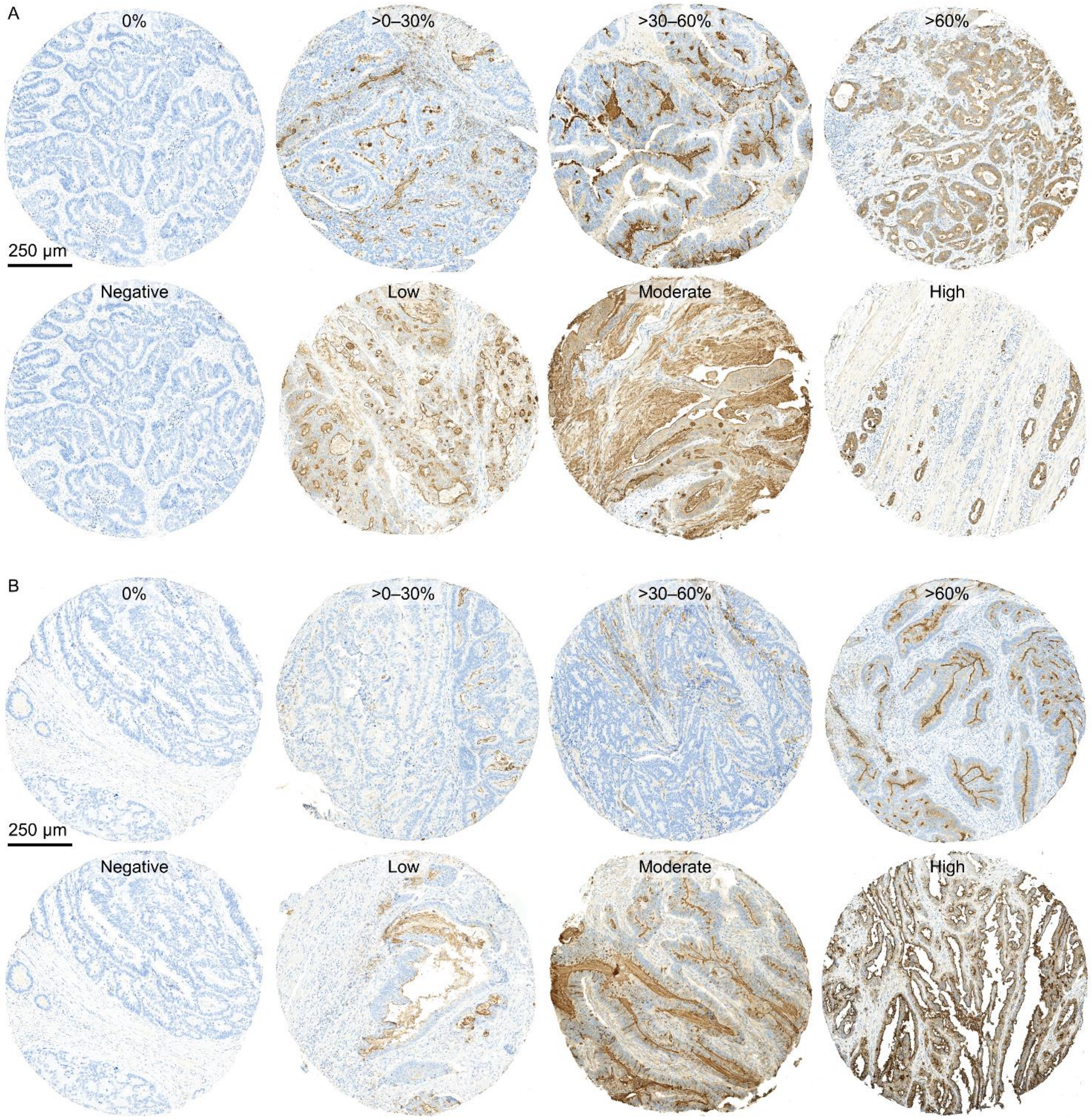

Figure S2. Staining patterns of cytoplasmic and membranous expression of MUC1. A, Example tumor cores representing cytoplasmic MUC1 expression levels (0–100%) and intensities (negative, low, moderate, and high). B, Example tumor cores representing membranous MUC1 expression levels (0–100%) and intensities (negative, low, moderate, and high).

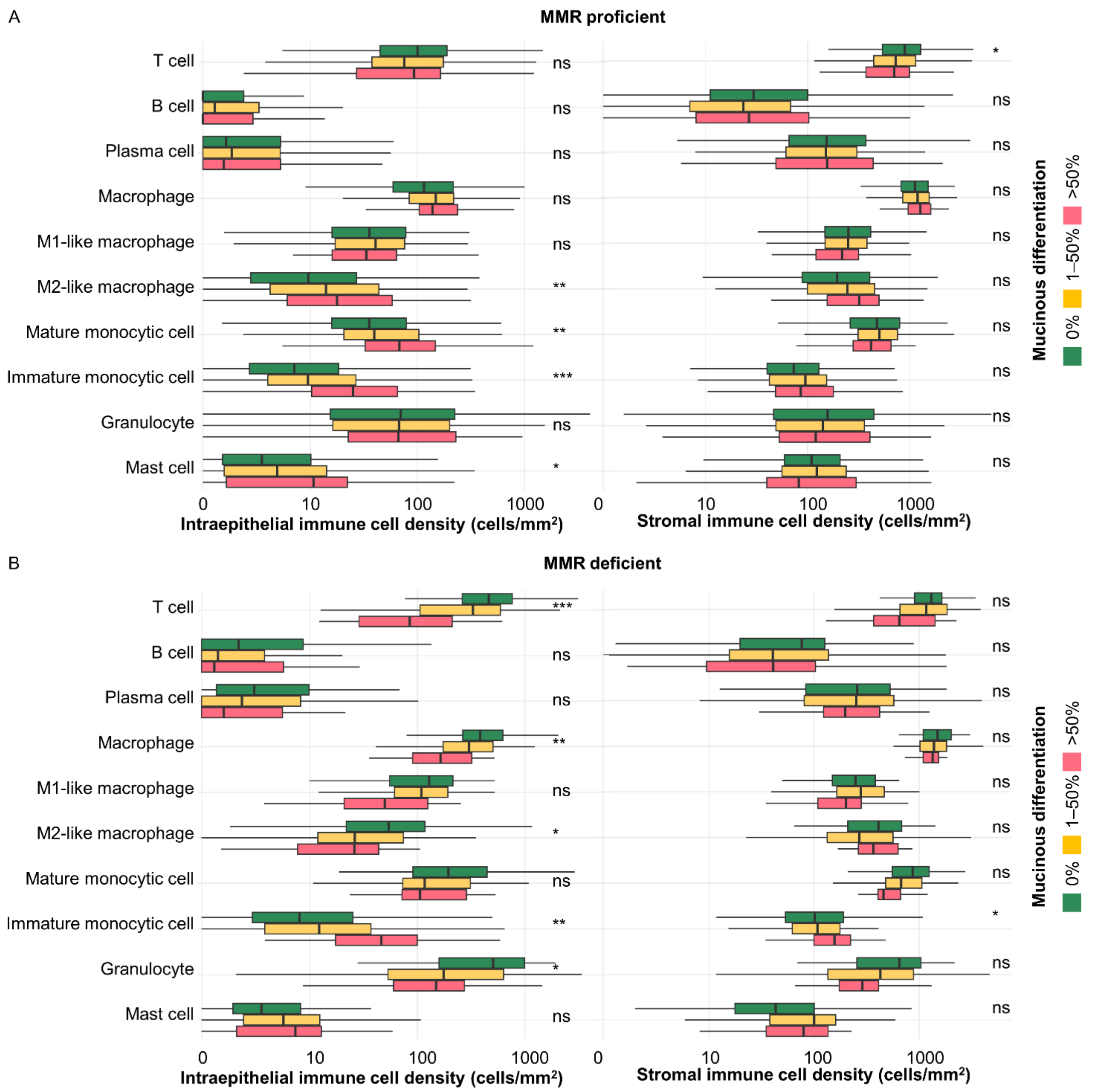

Figure S3. Associations between mucinous differentiation and intratumoral and stromal immune cell densities in MMR proficient (A) and deficient (B) tumors. *P* values were determined using the Wilcoxon rank-sum test. Statistically significant correlations are shown with asterisks (***, *P*<0.0001; **, *P*<0.001; *, *P*<0.005). MMR, mismatch repair. Immune cell density analyses for MMR proficient and deficient tumors are based on 870 and 156 cases (T cells, macrophages, M1-like macrophages, M2-like macrophages), 873 and 159 cases (B cells, plasma cells), 856 and 152 cases (mature monocytic cells, immature monocytic cells, granulocytes, mast cells), respectively. MMR, mismatch repair.

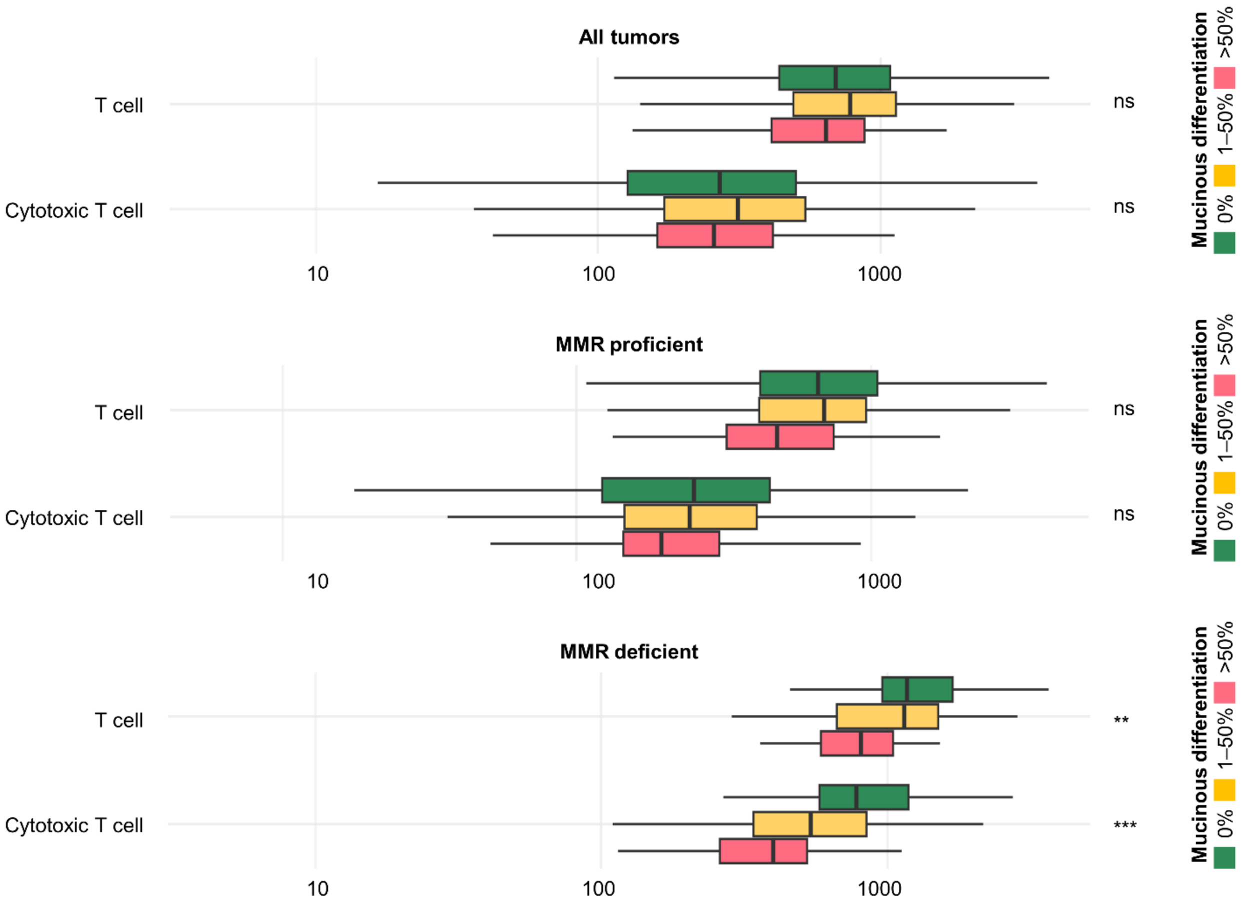

Figure S4. Associations between mucinous differentiation and immune cell densities in all tumors and separately in MMR proficient and deficient tumors in the validation cohort. *P* values were determined using the Wilcoxon rank-sum test. Statistically significant correlations are shown with asterisks (***, *P*<0.0001; **, *P*<0.001; *, *P*<0.005). MMR, mismatch repair.

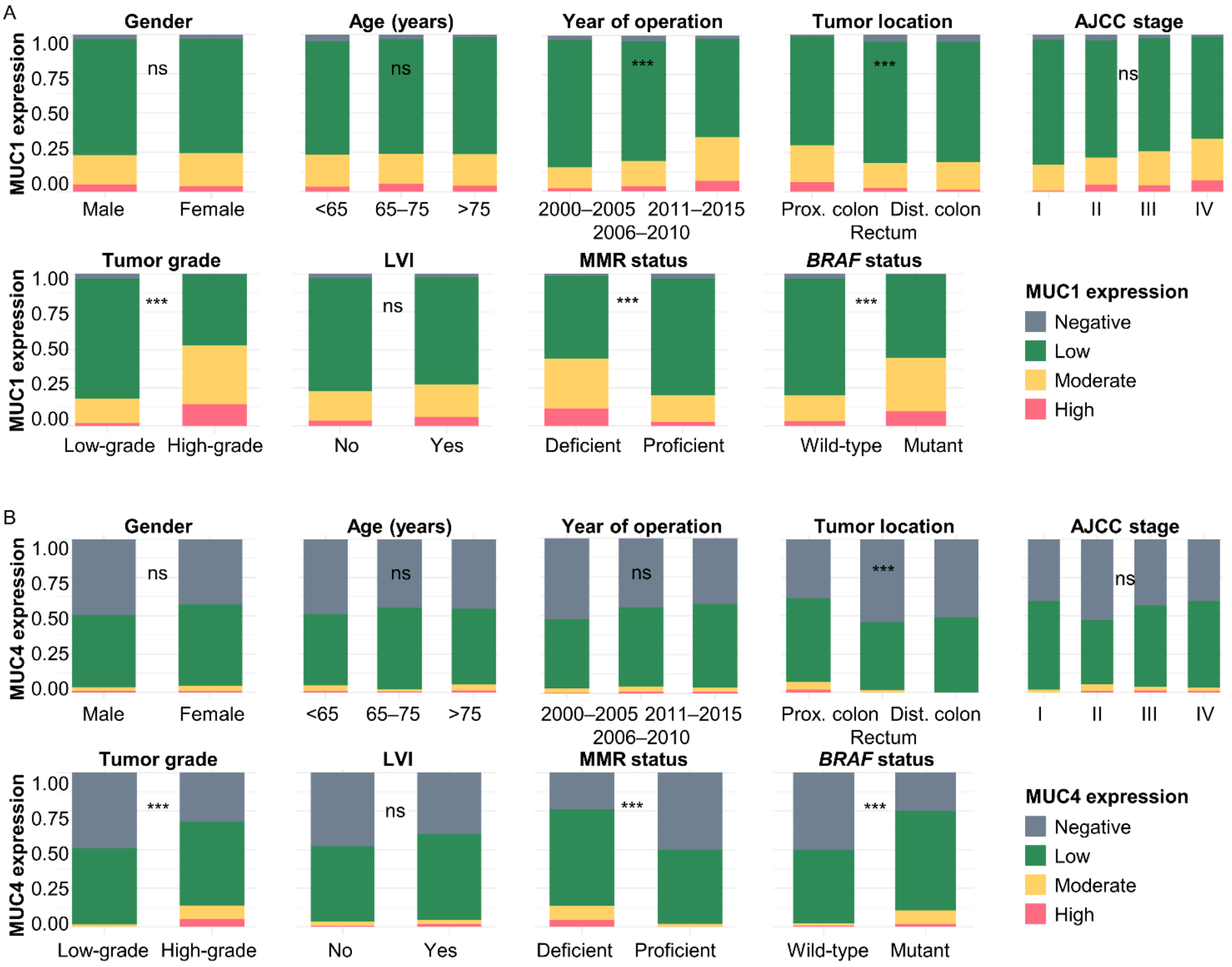

Figure S5. The associations between clinicopathological characteristics and cytoplasmic expression of transmembrane mucins Associations for A, MUC1 and B, MUC4 expression. *P* values were determined using the Chi-square test. Statistically significant correlations are shown with asterisks (***, *P*<0.0001; **, *P*<0.001; *, *P*<0.005). AJCC, American Joint Committee on Cancer; LVI, lymphovascular invasion; MMR, mismatch repair.

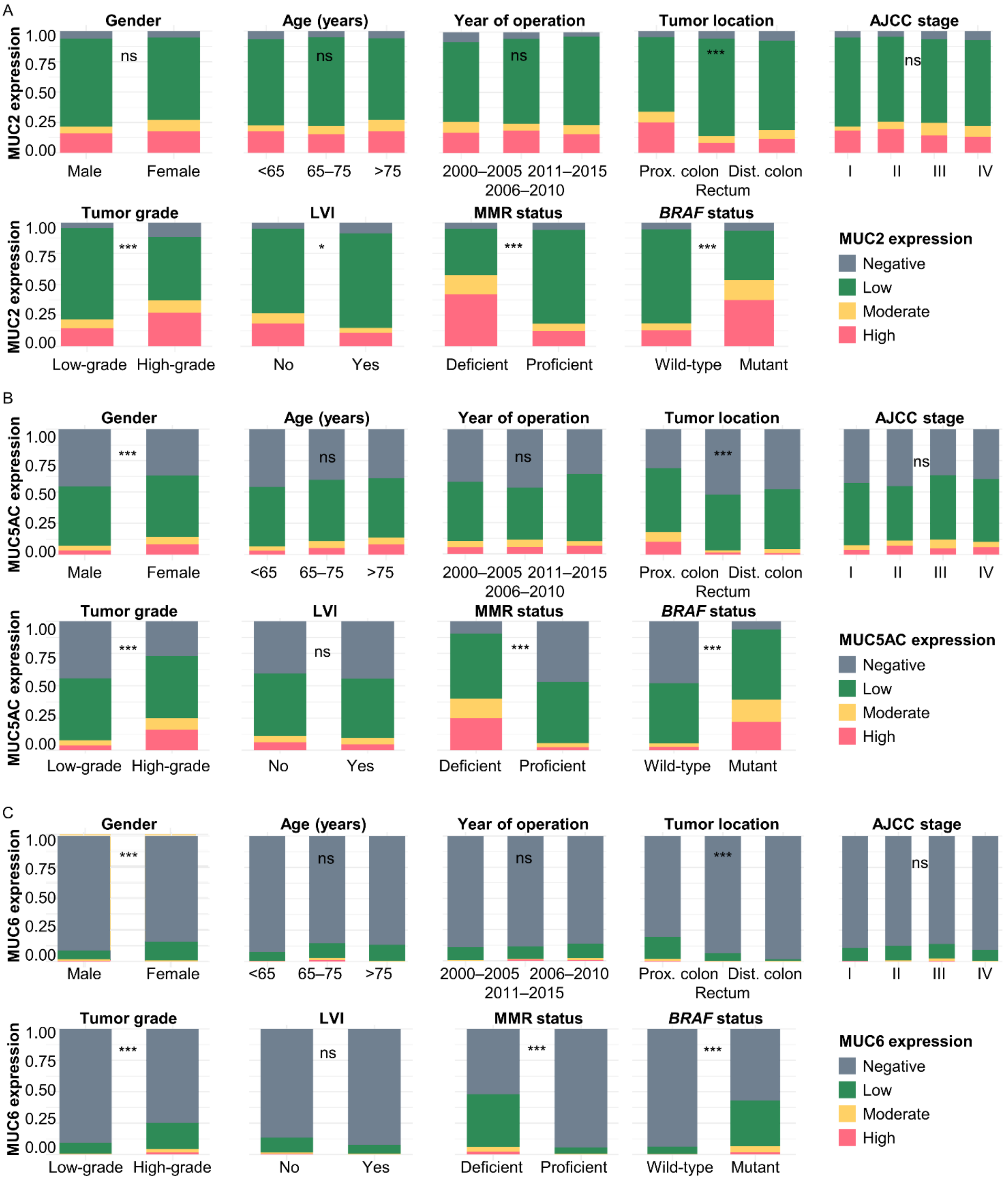

Figure S6. The associations between clinicopathological characteristics and cytoplasmic expression of secreted mucins. Associations for A, MUC2, B, MUC5AC, and C, MUC6 expression. *P* values were determined using the Chi-square test. Statistically significant correlations are shown with asterisks (***, *P*<0.0001; **, *P*<0.001; *, *P*<0.005). AJCC, American Joint Committee on Cancer; LVI, lymphovascular invasion; MMR, mismatch repair.

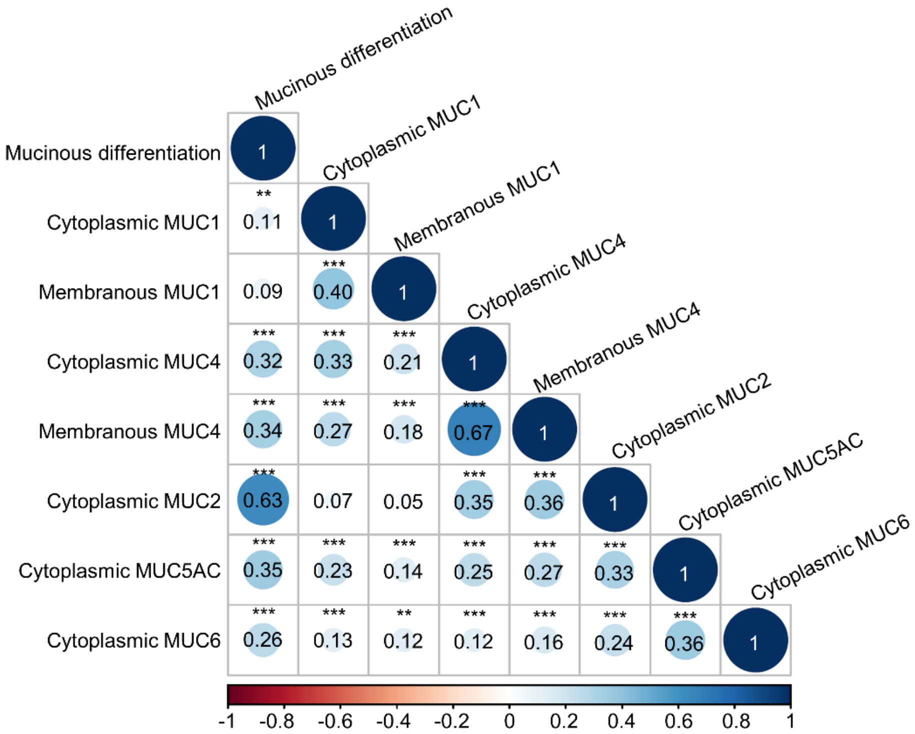

Figure S7. Spearman correlation coefficients between mucinous differentiation and the expression of mucins. Statistically significant correlations are shown with asterisks (***, *P*<0.0001; **, *P*<0.001; *, *P*<0.005). Mucinous differentiation represents the proportion of extracellular mucus within the tumor and was visually analyzed from H&E-stained whole slide tissue sections.

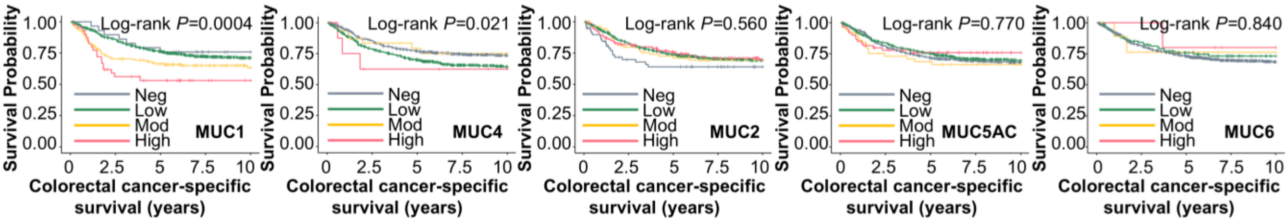

MUC1

	Years, number at risk				
	0	2.5	5	7.5	10
Neg	30	26	22	13	10
Low	768	610	490	348	237
Mod	209	132	111	69	36
High	42	20	15	5	2

MUC4

	Years, number at risk				
	0	2.5	5	7.5	10
Neg	484	384	320	230	149
Low	525	377	295	189	123
Mod	31	23	19	13	11
High	9	4	4	3	2

MUC2

	Years, number at risk				
	0	2.5	5	7.5	10
Neg	60	36	33	26	18
Low	735	564	457	303	197
Mod	78	56	47	32	20
High	176	132	101	74	50

MUC5AC

	Years, number at risk				
	0	2.5	5	7.5	10
Neg	433	328	259	191	132
Low	503	385	316	199	127
Mod	52	33	30	22	14
High	61	42	33	23	12

MUC6

	Years, number at risk				
	0	2.5	5	7.5	10
Neg	920	694	568	388	255
Low	114	84	62	43	26
Mod	10	5	4	2	2
High	5	5	4	2	2

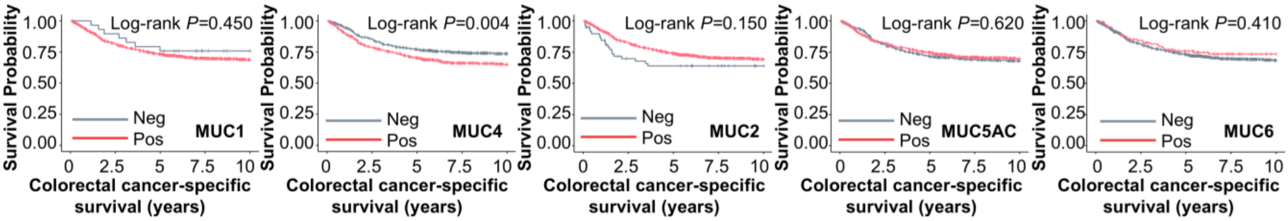

MUC1

	Years, number at risk				
	0	2.5	5	7.5	10
Neg	30	26	22	13	10
Pos	1019	762	616	422	275

MUC4

	Years, number at risk				
	0	2.5	5	7.5	10
Neg	484	384	320	230	149
Pos	565	404	318	205	136

MUC2

	Years, number at risk				
	0	2.5	5	7.5	10
Neg	60	36	33	26	18
Pos	989	752	605	409	267

MUC5AC

	Years, number at risk				
	0	2.5	5	7.5	10
Neg	433	328	259	191	132
Pos	616	460	379	244	153

MUC6

	Years, number at risk				
	0	2.5	5	7.5	10
Neg	920	694	568	388	255
Pos	129	94	70	47	30

Figure S8. Kaplan-Meier estimates for cancer-specific survival according to cytoplasmic expression of mucins. The expression of mucins is categorized into four groups (negative, low, moderate, high) and into negative and positive.

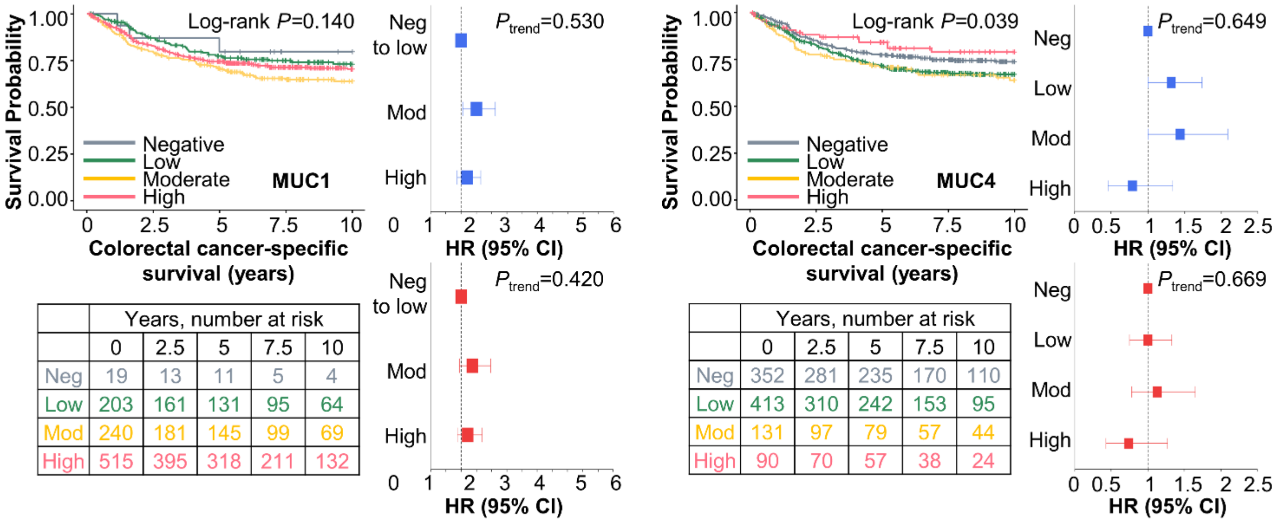

Figure S9. Kaplan-Meier survival curves and Cox proportion hazards regression models for cancer-specific survival for membranous expression of transmembrane mucins. Univariable (blue) and multivariable (red) Cox proportional hazards regression models are represented as forest plots with HRs along with their 95% CIs as whiskers. P_{trend} values were calculated by using the categories of mucin expression as continuous variables. Multivariable Cox proportional hazards regression models were adjusted for sex (male, female), age (<65, 65–75, >75), year of operation (2000–2005, 2006–2010, 2011–2015), tumour location (proximal colon, distal colon, rectum), stage (I–II, III, IV), tumour grade (low-grade, high-grade), lymphovascular invasion (negative, positive), MMR status (proficient, deficient), and *BRAF* status (wild-type, mutant). Mucin expression categories with fewer than 40 patients were combined with the adjacent ordinal category to enhance the stability of the multivariable regression models. CI, confidence interval; HR, hazard ratio; MMR, mismatch repair.

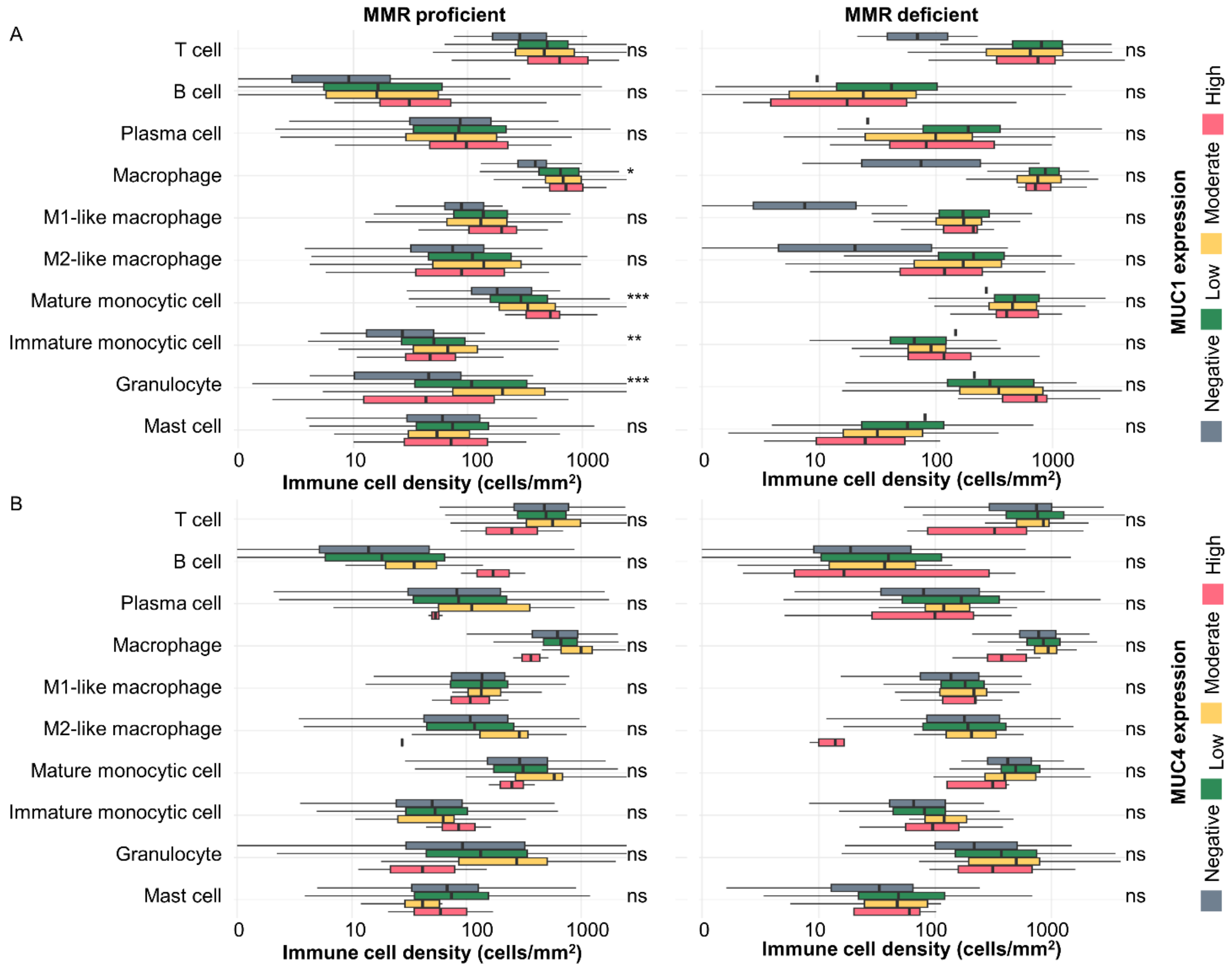

Figure S10. Associations between cytoplasmic expression of transmembrane mucins and immune cell densities in MMR proficient and deficient tumors. Associations for A, MUC1 and B, MUC4 expression. *P* values are determined using the Wilcoxon rank-sum test. Statistically significant correlations are shown with asterisks (***, *P*<0.0001; **, *P*<0.001; *, *P*<0.005). Immune cell density analyses for MMR proficient (N=889) and deficient (N=160) tumors are based on 870 and 156 cases (T cells, macrophages, M1-like macrophages, M2-like macrophages), 873 and 159 cases (B cells, plasma cells), 856 and 152 cases (mature monocytic cells, immature monocytic cells, granulocytes, mast cells), respectively. MMR, mismatch repair.

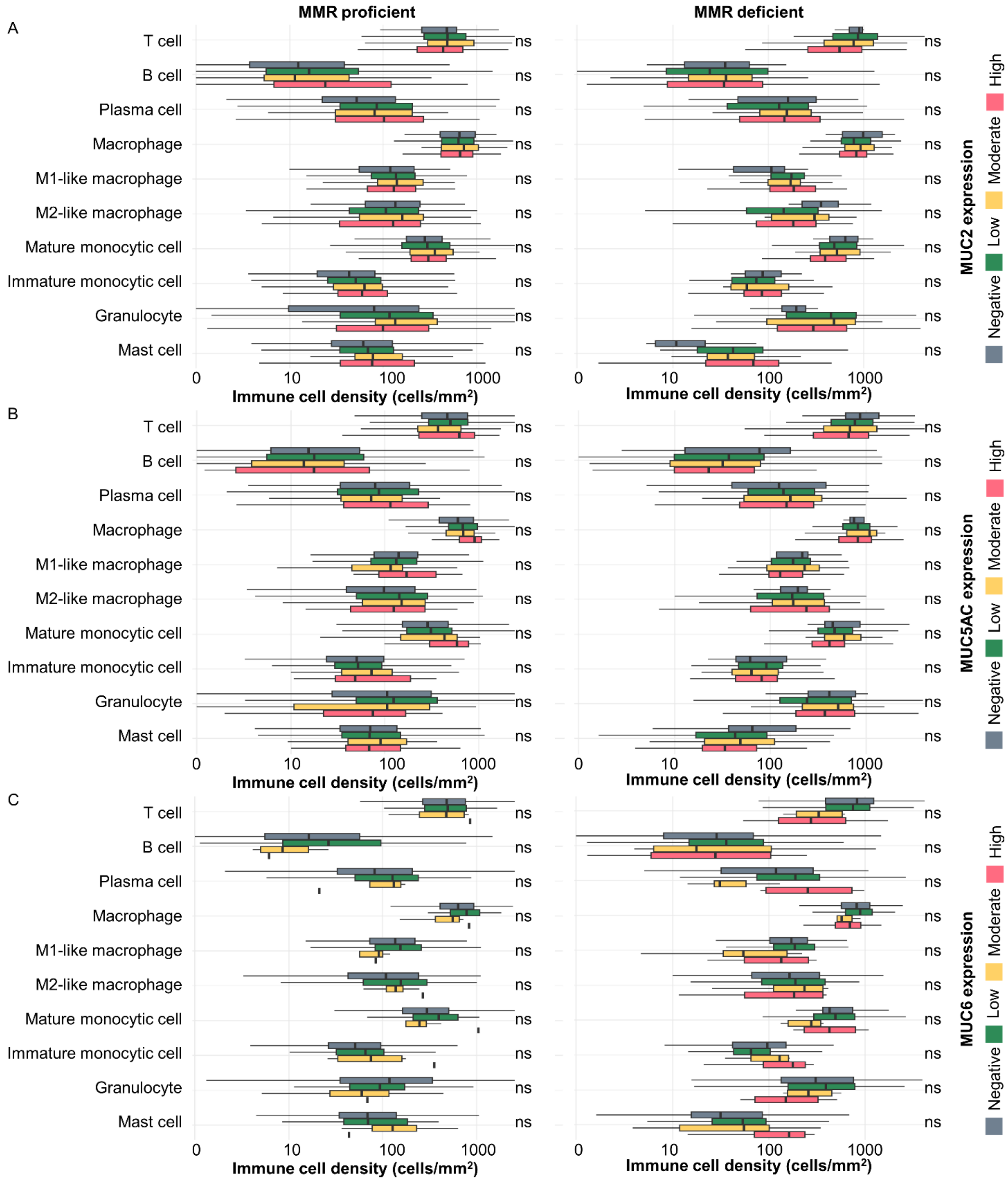

Figure S11. Associations between cytoplasmic expression of secreted mucins and immune cell densities in MMR proficient and deficient tumors. Associations for A, MUC2 and B, MUC5AC, and C, MUC6 expression. *P* values are determined using the Wilcoxon rank-sum test. Statistically significant correlations are shown with asterisks (***, *P*<0.0001; **, *P*<0.001; *, *P*<0.005). Immune cell density analyses for MMR proficient (N=889) and deficient (N=160) tumors are based on 870 and 156 cases (T cells, macrophages, M1-like macrophages, M2-like macrophages), 873 and 159 cases (B cells, plasma cells), 856 and 152 cases (mature monocytic cells, immature monocytic cells, granulocytes, mast cells), respectively. MMR, mismatch repair.
